# Tertiary lymphoid structures and B-cell infiltration are IPF features with functional consequences

**DOI:** 10.3389/fimmu.2024.1437767

**Published:** 2024-10-11

**Authors:** Elisabetta Cocconcelli, Elisabetta Balestro, Graziella Turato, Giordano Fiorentù, Erica Bazzan, Davide Biondini, Mariaenrica Tinè, Nicol Bernardinello, Federica Pezzuto, Simonetta Baraldo, Fiorella Calabrese, Federico Rea, Alessandro Sanduzzi Zamparelli, Paolo Spagnolo, Manuel G. Cosio, Marina Saetta

**Affiliations:** ^1^ Department of Cardiac, Thoracic, Vascular Sciences and Public Health, University of Padova, Padova, Italy; ^2^ Department of Medicine, University of Padova, Padova, Italy; ^3^ Respiratory Medicine Unit at the Monaldi Hospital, AO dei Colli, Department of Clinical Medicine and Surgery, Federico II University, Naples, Italy; ^4^ Meakins-Christie Laboratories, Respiratory Division, McGill University, Montreal, QC, Canada

**Keywords:** autoimmunity, B cell, early IPF, IPF progression, CD40

## Abstract

**Background:**

Recent literature has shown the presence of B cells and autoantibodies in idiopathic pulmonary fibrosis (IPF) which would imply the presence of tertiary lymphoid structures (TLS, sites where the immune response is triggered), yet TLS are not considered features of the histological characteristics of IPF.

**Aim:**

This study aims to quantify the presence, size, and degree of activation of TLS in biopsied and explanted lungs from patients with early- and late-IPF, never treated with antifibrotics, and relate their presence and activity to the clinical course, disease progression, and lung inflammation.

**Methods:**

Immunohistochestry for B cells and CD4, CD8, and CD45 cells was performed in lung tissue from IPF patients: 18 at diagnosis (early), 39 explanted (end-stage), and 12 smoking controls. TLS activation was assessed by CD40 expression. Spirometry along 31 (12–72) months of follow-up was used to characterize end-stage IPF as slow progressors or rapid progressors.

**Results:**

B cells, along with other inflammatory cells, were higher in early- and end-stage IPF than in controls (*p* < 0.001). In rapid progressors, all inflammatory cells were higher than in slow progressors (*p* < 0.05). TLS were present in 100% of early- and end-stage IPF and in 50% of controls. In end-stage IPF, the TLS area and activation score were higher than in early IPF (*p* < 0.0001; *p* = 0.005) and controls (*p* < 0.04; *p* < 0.002). TLS activation score correlated with FVC decline during follow-up in rapid progressors (*r* = 0.73; *p* = 0.007) but not in slow progressors.

**Conclusions:**

A prominent B-cell infiltration, along with the presence of TLS, the activity of which correlates with FVC decline, is an important component of IPF from the beginning of the disease, likely playing an important role on its mechanism and progression.

## Introduction

Idiopathic pulmonary fibrosis (IPF) is defined as a specific form of progressive fibrosing interstitial pneumonia of unknown cause, limited to the lungs and associated with the histopathology of usual interstitial pneumonia (UIP). The diagnosis of IPF requires the exclusion of other forms of interstitial pneumonias, including other idiopathic interstitial pneumonias and interstitial lung diseases (ILD) associated with environmental exposures, medications, and connective tissue disease. The histological pattern of UIP is defined as dense fibrosis with architectural distortion, patchy involvement of lung parenchyma by fibrosis, fibroblast foci, and, importantly, absence of features like cellular inflammatory infiltrates away from areas of honeycombing and prominent lymphoid hyperplasia, including germinal center, which suggest alternative diagnosis pointing toward other types of fibrosis (1).

The strict differentiation of IPF from other ILDs has been postulated as essential to the point of recommending lung biopsy when HRCT diagnosis of UIP is uncertain since a proper diagnosis of IPF is necessary for its treatment with the antifibrotic drugs pirfenidone and nintedanib. However, recent publications have cast doubts about the importance of the strict separation of the different types of ILDs. An analysis of the clinical course of IPF [INPULSIS trial (2)] and other ILDs [INBUILD trial (3)] suggests that progressive ILDs other than IPF not only have clinical courses similar to IPF but also responded to nintedanib treatment by significantly decreasing FVC decay independently of the fibrotic pattern on HRCT (3–5). These data support the hypothesis that “progressive interstitial lung diseases, regardless of clinical diagnosis, have a similar pathobiological mechanism which might represent a common fibrotic response to tissue injury” (6–9).

Recent literature has shown that adaptive inflammation consisting of CD4+, Cd8+, CD68+, B cells and TLS is present in the lung of IPF (10, 11) and that B cells, plasma cells, and autoantibodies in IPF might have a specific role in disease pathogenesis (12). These findings replicate findings in other types of ILDs in which treatment with anti-CD20 antibodies against B cells has been successfully used (13). The presence of B and plasma cells along with antigen–antibody complexes in IPF would go along with the presence of tertiary lymphoid structures (TLS) with germinal centers (14, 15). These structures have been described previously in IPF lungs (16, 17), but the presence of TLS is not considered as part of the histological characteristics of IPF (1).

It was our aim to investigate and morphometrically quantify the presence of inflammatory cells and TLS in lungs from patients with clinical and pathological diagnosis of IPF at the early (at diagnosis) and late (at transplantation) stages of disease and relate their presence to the disease progression.

Our aim would be to address the recently stated concern for a “neglect of contributions of immune cells to the pathogenesis of fibrotic ILDs, including IPF” and the consequent exhortation for “an urgent need for further investigation and characterization of immune cell populations within ILD lungs” (18). For that purpose, we studied an IPF population with early disease diagnosed with surgical lung biopsies and explanted lungs from patients with end-stage IPF who had never been treated with antifibrotic medications.

## Methods

### Study population

The study population consisted of 18 patients with IPF who underwent video-assisted thoracic surgery (VATS) biopsy as part of the initial diagnostic evaluation (early IPF) and 39 patients with IPF who underwent lung transplant (end-stage IPF). A total of 12 smokers with normal lung function, undergoing lung resection for nodules, were included as the control group. Early-IPF patients were recruited at the Interstitial Lung Disease Center of Padova (*n* = 7) and Napoli (*n* = 11) between 2014 and 2018, while end-stage IPF patients belonged to a cohort who underwent lung transplantation at the Padova center between 2011 and 2014, before the advent of the antifibrotic therapy (19). Furthermore, 70% of the end-stage IPF patients were treated with prednisone with or without azathioprine according to the 2000 guidelines (20). IPF was diagnosed according to the ATS/ERS/JRS/ALAT guidelines (20, 21), and none of the subjects had a history of occupational or environmental exposure nor clinical features of hypersensitivity pneumonitis or connective tissue disease. As part of the initial interstitial lung disease accepted protocol for the diagnosis of IPF, the exclusion of other diagnosis such as undifferentiated connective tissue disease (CTD) or interstitial pulmonary diseases with autoimmune features (IPAF) is needed. Accordingly, all patients were screened for circulating anti-nuclear antibody (ANA), rheumatoid factor (RF), and anti-cyclic citrullinated peptide antibodies (anti-CCP), all of which were not elevated at significant levels. Because the group of patients in our study was investigated years before the new immunological concepts in the pathogenesis of IPF, including the description of autoantibodies against the intermediate filament protein periplakin (PPL) in patients with IPF, were known, these autoantibodies were not studied.

Spirometry was serially performed every 6 to 12 months in the end-stage patients during follow-up (from diagnosis to transplant). The fall in % predicted FVC per year was used to characterize the disease progression as “rapid” (fall in % predicted FVC >10%/year) and “slow” (fall in % predicted FVC < 10%/year) (19).

The study was performed in accordance with the Declaration of Helsinki and approved by the Ethics Committee of the University Hospital of Padova (4280/AO/17). Informed consent was obtained for all study participants before surgery. None of the transplant donors was from a vulnerable population, and all donors or next of kin provided written informed consent that was freely given.

### Pathological analysis

Biopsies and lung samples were fixed in formalin and embedded in paraffin. Then, 5-μm-thick sections were obtained and stained for immunohistochemical analysis as previously described (19). The diagnosis of IPF was confirmed by the presence of a usual interstitial pneumonia (UIP) pattern by an expert pathologist (FC) (20, 21). B lymphocytes (CD20+), T lymphocytes (CD4+ and CD8+), and total leukocytes (CD45+) were identified by immunohistochemical analysis and quantified by one observer throughout the parenchyma and expressed as the number of positive cells per square millimeter of lung tissue as previously done (19) (details in **Supplementary Materials**).

**Figure 1:**
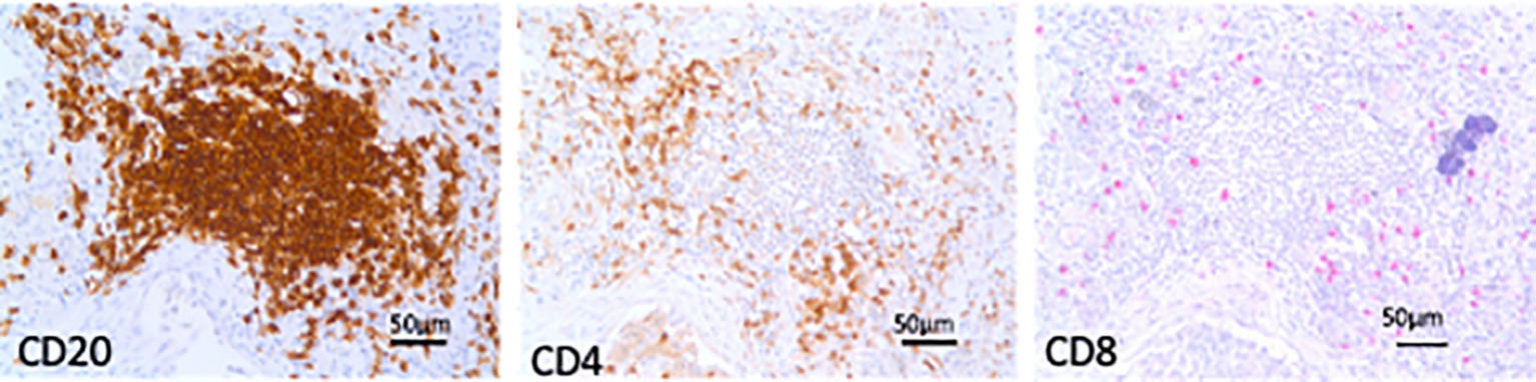
Tertiary lymphoid structures (TLS). Microphotographs of TLS in sequential lung sections from an end-stage idiopathic pulmonary fibrosis subject showing aggregates of more than 40 mononuclear cells exhibiting the topographical arrangement of CD20+ B cells surrounded by CD4+ and CD8+ T cells. Bars = 50 micrometers.

TLS, aggregates of more than 40 mononuclear cells exhibiting the topographical arrangement of CD20+ B cells surrounded by CD4+ and CD8+ T cells with a germinal center (22), were identified, counted, and measured on lung sections stained for C20+ B lymphocytes and expressed as the number of TLS per square centimeter of lung tissue examined and as TLS mean area in square micrometers (**Figure 1**).

The TLS state of activation was graded by the extent of the expression of the co-stimulatory and activation marker CD40 (23) on TLS using a semiquantitative score, ranging from 0 to 4 as follows: 0 (no CD40 expression), 1 (between 1% and 25%), 2 (between 26% and 50%), 3 (between 51% and 75%), and 4 (>76%). In each case, the CD40 activation scores of all TLS were totaled and expressed as percentage of the maximum computable score for the case (24).

### Statistical analysis

The patients’ characteristics were described using absolute number and percentage for categorical variables and median and range for continuous variables. Differences between groups were evaluated with Kruskal–Wallis test and Mann–Whitney *U*-test. Distributions of categorical variables was investigated by using *χ* ^2^. Correlation coefficients were calculated using the nonparametric Spearman rank method. *P*-values of 0.05 or less were considered to indicate statistical significance. All data were analyzed using SPSS software, version 25.0 (New York, NY, USA: IBM Corp., USA). The statistical package GraphPad Prism 7.0 (GraphPad Software, Inc., La Jolla, CA, USA) was used for graphs.

## Results

### Clinical and functional characteristics at baseline

The clinical and functional characteristics of early IPF, end-stage IPF, and smoking controls are shown in **Table 1**. The majority of subjects were male. Moreover, 65% of IPF subjects were smokers but smoked less than the control smokers. The FVC % predicted at diagnosis was significantly lower in end-stage than in early IPF, while DLCO was similarly decreased in the early and end stages.

**Table 1:** Clinical and functional characteristics at diagnosis of early IPF, end-stage IPF, and smoking controls.

	Early IPF (*n* = 18)	End-stage IPF (*n* = 39)	Smoking controls (*n* = 12)	*P-*values
**Male, *n* (%)**	14 (78)	30 (77)	11 (92)	n.s.
**Age, years**	58 (54 - 61)^a^	53 (33 - 64)^a^	66 (56 - 82)	P<0.001
**Smokers, *n* (%)**	11 (61)	27 (69)	12 (100)	n.s.
**Smoking history, pack-years**	4 (0 - 30)^a^	15 (0 - 120)^a^	49 (15 - 102)	P<0.001
**FEV_1_, L**	3.06 (1.87 – 3.58)	2.46 (0.7 – 4.1)	2.39 (1.54 – 3.54)	n.s.
**FEV_1_, % pred.**	81 (51 - 118)^a,^ b	62 (26 - 93)^a^	98 (74 - 116)	P<0.001
**FVC, L**	2.96 (1.45 - 4.06)	2.4 (0.7 – 4.06)^a^	3.02 (1.75 – 4.60)	P<0.01
**FVC, % pred.**	75 (40-109)^a,b^	61 (20 - 93)^a^	123 (90 - 142)	P<0.001
**FEV_1_/FVC, %**	98 (75 - 131)^a^	102 (80 - 125)^a^	80 (74 - 91)	P<0.01
**DLCO, %pred.**	39 (16 - 74)^a^	36 (10 - 85)^a^	81 (75 - 91)	P<0.01

Values are expressed as numbers and (%) or median and (range) as appropriate.

n.s., non-significant.

a Significantly different from smoking controls.

b Significantly different from end-stage IPF.

### Pathological analysis: Tertiary lymphoid structures (TLS) and inflammatory cells in lung tissue

#### Tertiary lymphoid structures

TLS in lung parenchyma were present in all subjects with early and end-stage IPF but only in 50% of smoking controls. Numerous CD4+ and CD8+ T cells surrounded the TLS with a CD4/CD8 ratio similar to the ratio found in the lung parenchyma (**Supplementary Figure S1**).

**Figure 2:**
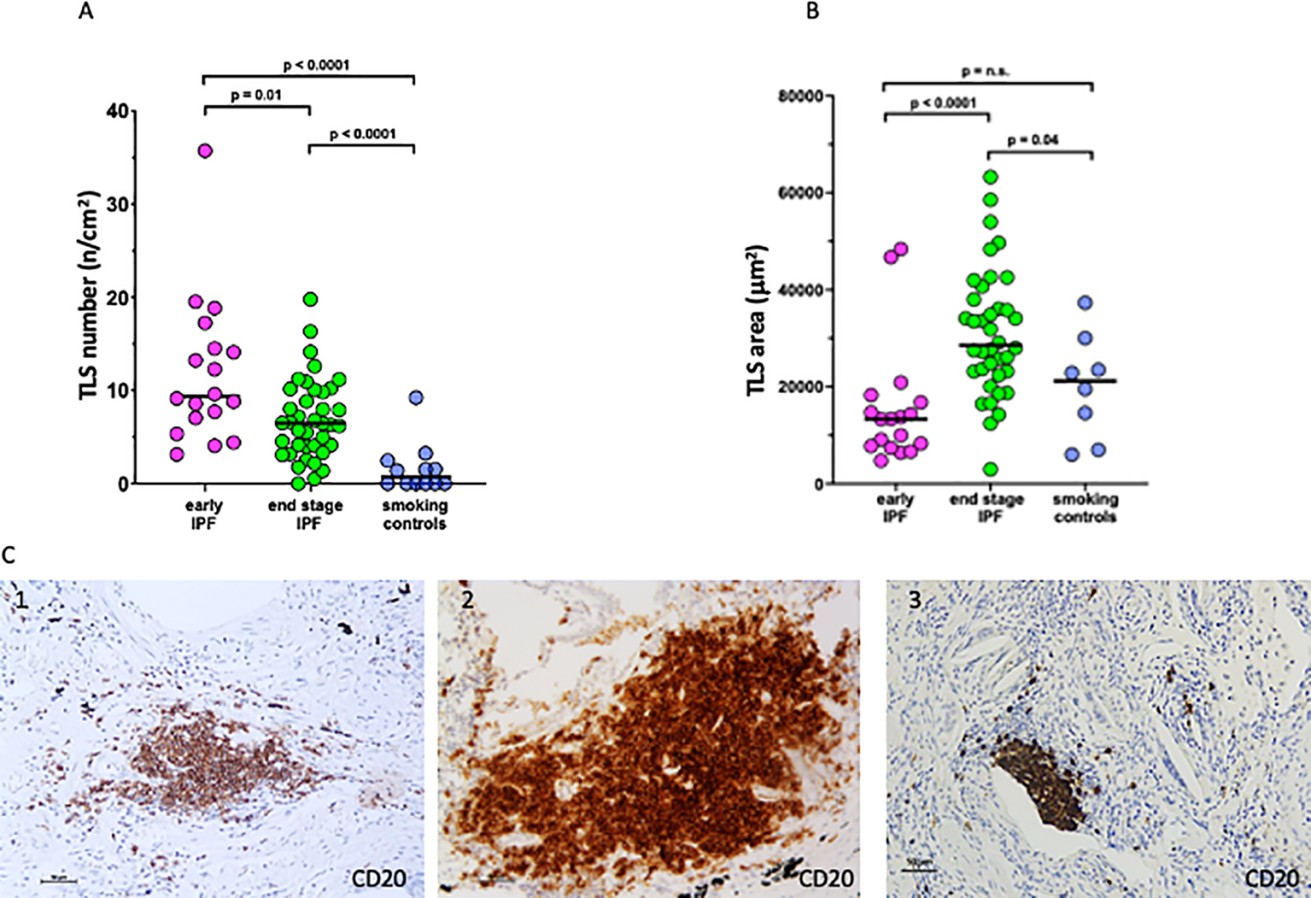
Number and size of tertiary lymphoid structures (TLS). **(A)** Number of TLS in lung tissue in early idiopathic pulmonary fibrosis (IPF), end-stage IPF, and smoking controls. **(B)** Mean area of TLS in lung tissue in early IPF, end-stage IPF, and smoking controls. Overall comparison by Kruskal–Wallis test (*p* < 0.01 for all). **(C)** Microphotographs of TLS in a patient with early IPF (1), end-stage IPF (2), and smoking control (3) showing aggregates of more than 40 mononuclear cells exhibiting the topographical arrangement of CD20+ B cells. Bars = 50 micrometers.

The number of TLS per square centimeter of lung tissue (**Figures 2A, C**) was higher in the early than in end-stage IPF [9 (3–36) vs. 6 (0.5–16) TLS/cm^2^; *p* = 0.01], and both were higher than in the smoking controls [0.7 (0–9); *p* < 0.0001]. By contrast, the mean area of TLS per case was higher in end-stage than in early IPF and smoking controls [27,974 (12,595–58,098) vs. 13,329 (4,724-48,418) vs. 21,184 (6,012–37,323 um^2^); *p* < 0.0001 and *p* = 0.04; **Figures 2B, C**].

**Figure 3:**
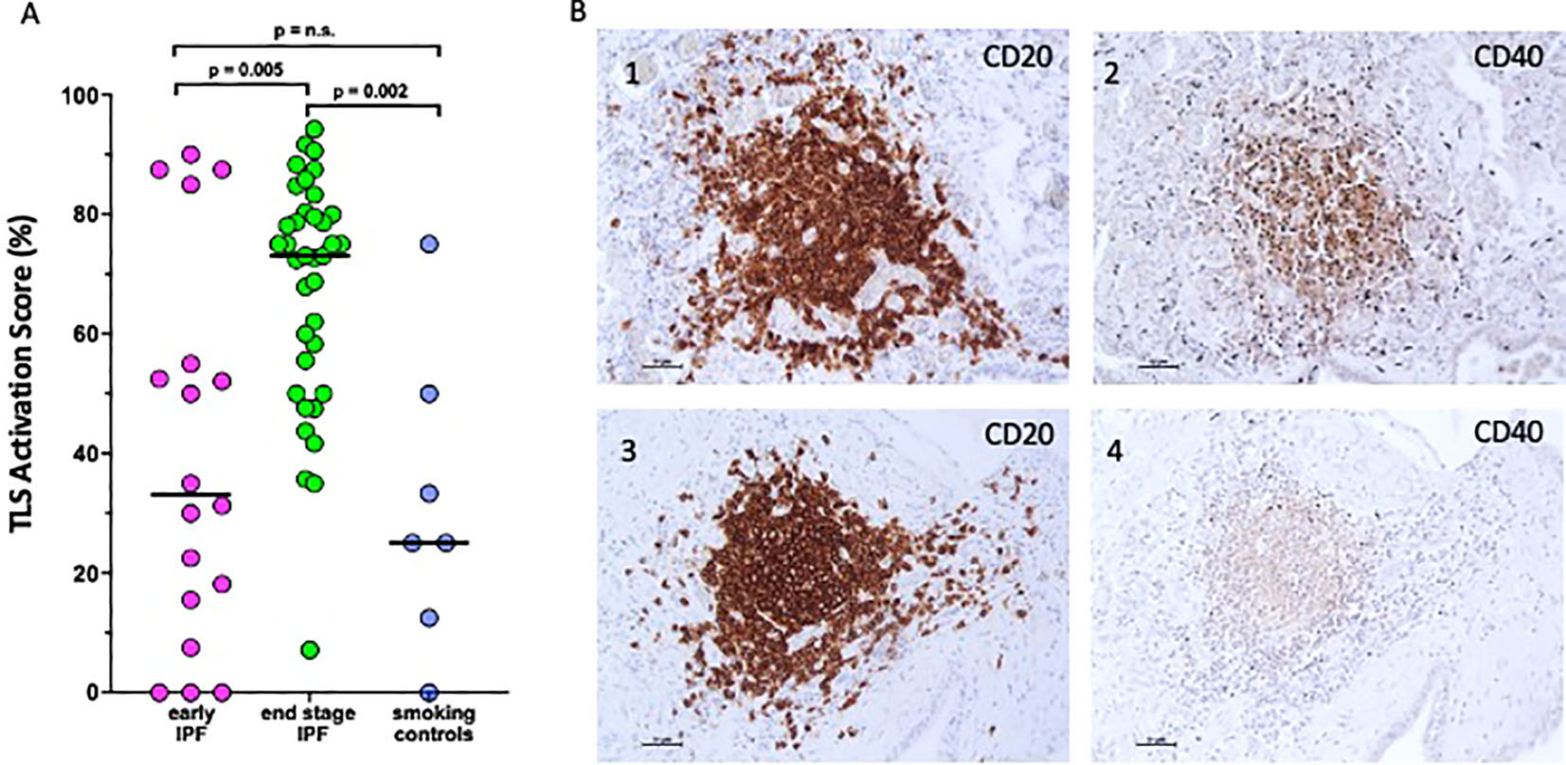
Activation score of tertiary lymphoid structures. **(A)** CD40 activation score expressed as a percentage of the maximum computable score for each patient in early idiopathic pulmonary fibrosis (IPF), endstage IPF, and smoking controls. Horizontal bars represent median values. Overall comparison by Kruskal–Wallis test (p < 0.01 for all). **(B)** Microphotograph showing CD20 expression (1, 3) and CD40 expression (2, 4) in the same TLS.

Furthermore, 98% of TLS were activated (CD40 positive staining) in end-stage IPF compared to the 55% in early IPF. In end-stage IPF, the activation score (CD40+) was higher than in early IPF and smoking controls [73 (9–92) vs. 33 (0–90)%; *p* = 0.005 vs. 23 (0–75); *p* = 0.002], which suggests a possible role of TLS activation in the deterioration of pathology. In early IPF, the activation score was not different than in smoking controls (**Figure 3A**).

**Figure 4:**
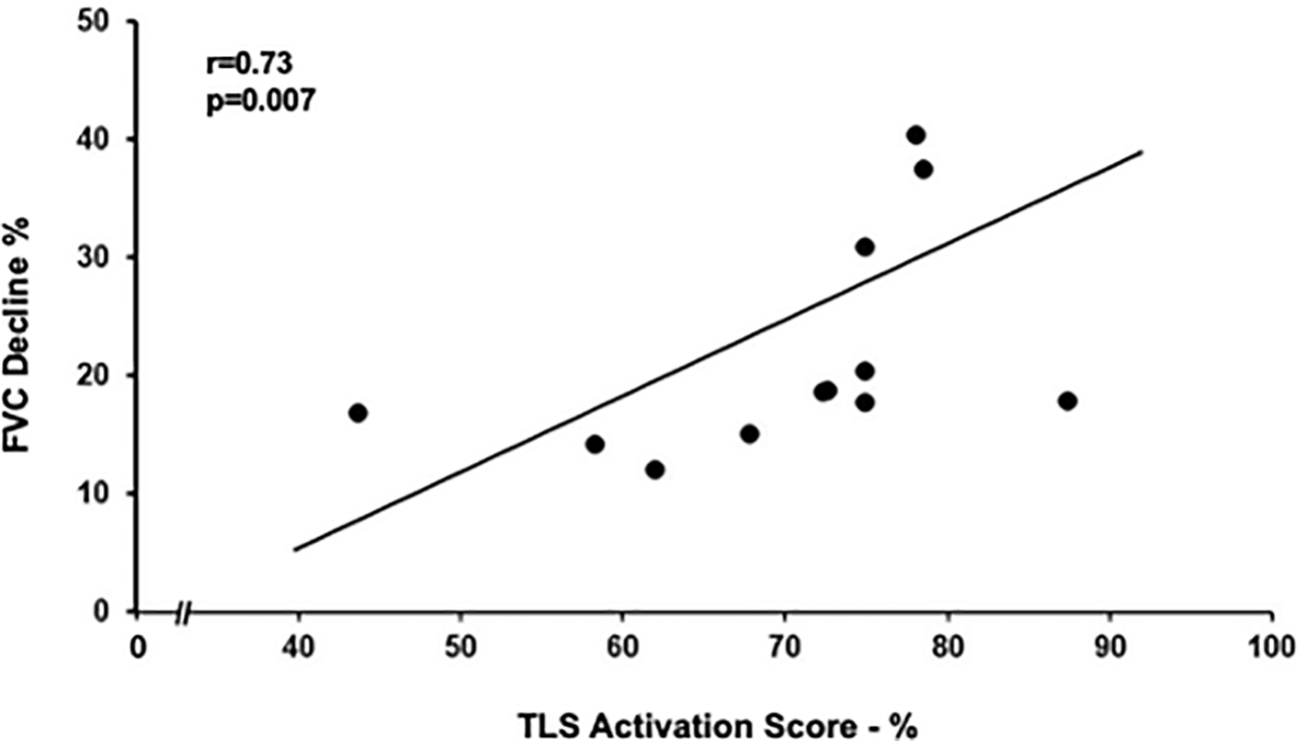
Relation between tertiary lymphoid structures (TLS) activation score and FVC decline in rapid progressors. TLS activation score positively correlated with yearly decline in FVC (as % of FVC at diagnosis). Spearman rank correlation (r = 0.73 and p = 0.007).

CD20 (a B-cell-specific membrane protein and pan-B cell marker, identifying all B cells regardless of their activation status) was strongly positive in TLS (**Figures 3B, 1** and **3**), while CD40 (a receptor primarily expressed on activated B cells and not universally expressed across all B cells) was variable and not consistently positive in the same TLS (**Figures 3B, 2** and **4**), highlighting a subset of these B cells that are activated.

When all cases were examined together, TLS number, area, and activation score correlated weakly but significantly to FVC % predicted at diagnosis (*r* = -0.3, *p* = 0.016; *r* = -0.28, *p* = 0.03; and *r* = -0.28, *p* = 0.03, respectively).

#### Inflammatory cells

The total leukocyte numbers (CD45+ cells) in lung parenchyma were similar in early and end-stage IPF [390.5 (216–733) vs. 380 (149–789) cells/mm^2^] but higher than in smoking controls [112 (37–237); *p* < 0.0001 for both; **Table 2**]. The number of CD45+/mm^2^ in all cases together correlated with the number of TLS/cm^2^ (*r* = 0.4, *p* = 0.001).

**Table 2:** Inflammatory cells in the lung parenchyma of early IPF, end-stage IPF, and smoking controls.

	Early IPF (*n* = 18)	End-stage IPF (*n* = 39)	Smoking controls (*n* = 12)	*P*-values
**CD45/mm^2^**	390.5 (216–733)^a^	380 (149–789)^a^	112 (37–237)	*P* < 0.001
**CD8/mm^2^**	92.8 (16.3–184.4)^a,b^	44 (7.6–196)	62.9 (5.4–160.7)	*P* = 0.016
**CD4/mm^2^**	191.5 (40–689.7)	150 (20–563.8)	124.6 (52.8–321.5)	n.s.
**CD20/mm^2^**	46.0 (16.0–75.0)^a^	59.4 (3.4–389)^a^	25.6 (14.0–38.0)	*P* < 0.001

Values are expressed as median (range).

n.s., non-significant.

a Significantly different from smoking controls.

b Significantly different from end-stage IPF.

The number of CD4+ cells per square millimeter was similar in early IPF [191 (40–689) cells/mm^2^], end-stage IPF [150 (20–564) cells/mm^2^], and smoking controls [125 (53–321) cells/mm^2^; **Table 2**, **Supplementary Figure S2**]. The number of CD8+ cells per square millimeter was higher in early than in end-stage IPF [93 (16–184) cells/mm^2^ vs. 44 (7.6–196) cells/mm^2^; *p* = 0.006] and smoking controls [63 (5.4–161) cells/mm^2^; *p* = 0.03; **Table 2**, **Supplementary Figure S2**]. The number of CD20+ cells per square millimeter was similar in both early and end-stage IPF [46 (16–75) cells/mm^2^ vs. 59.4 (3.4–389) cells/mm^2^] but higher than in smoking controls [25.6 (14–38) cells/mm^2^; *p* < 0.005 for both]. All inflammatory cells were diffusely present throughout the lung parenchyma (**Supplementary Figure S2**).

There were no differences in any of the morphometric parameters described when smokers and never smokers with IPF were compared.

### Disease progression in end-stage IPF population: slow and rapid progressors

The median FVC decay of the end-stage IPF during a follow-up time of 34 ± 15 months [31 (12–72)] was 0.17 (0–1.1) L/year or 5 (0-27) % predicted/year. Based on the FVC decline, cases were classified as slow progressors (less than 10% predicted/year—27 cases or 69% of the population) and rapid progressors (greater than 10% predicted/year—12 cases or 31% of the population), the clinical characteristics of which are shown in **Table 3**.

**Table 3:** Clinical and functional characteristics of end-stage IPF categorized as slow and rapid progressor.

	Slow progressor (*n* = 27)	Rapid progressor (*n* = 12)	*P*-values
**Male, *n* (%)**	20 (74)	10 (83)	n.s.
**Age, years**	53 (40–64)	52 (33–64)	n.s.
**Smokers, *n* (%)**	18 (66)	9 (75)	n.s.
**Smoking history, pack-years**	14 (0–120)	17.5 (0–92)	n.s.
**FEV_1_, L**	1.8 (0.7–3.6)	2.9 (1.8–4.1)	*P* < 0.009
**FEV_1_, pred.**	54 (26–93)	71.5 (56–85)	*P* < 0.002
**FVC, L**	1.5 (0.7–2.7)	1.8 (1.1–2.5)	*P* < 0.01
**FVC, % pred.**	57 (20–93)	71 (56–86)	*P* < 0.008
**FEV_1_/FVC, %**	103 (80–125)	100 (97–110)	n.s.
**DLCO, %pred.**	37 (15–79)	36 (10–85)	*P* < 0.01
**Decline FVC, L per year**	0.05 (0–0.3)	0.48 (0.3–1.4)	*P* < 0.0001
**Decline FVC, % per year**	1.5 (0–9)	11 (10–28)	*P* < 0.0001
**Follow-up, months**	41 (12–72)	30 (12–46)	*P* < 0.01

Values are expressed as median (range) or numbers (%) as appropriate.

n.s., non-significant.

FVC decline/year was 0.05 (0–0.3) L/year or 1.5 (0-9) % /year in the slow and 0.48 (0.3–1.4) L/year or 11 (10-28) % /year (*p* < 0.0001 for both) in the rapid progressors, while the FVC at diagnosis was lower in the slow 1.5 (0.7–2.7) L or 57% predicted than in the rapid progressors [1.8 (1.1–2.5) L (*p* < 0.01) or 71% predicted (*p* < 0.008)]. The rate of progression, both in L/year (*r* = 0.6, *p* < 0.001) and predicted/year (*r* = 0.5, *p* < 0.001), of the whole end-stage group was significantly correlated with the FVC at diagnosis, indicating that the smaller the initial FVC, the slower the progression.

The rapid progressors had a significantly higher number of CD45, CD8, and CD4 inflammatory cells per square millimeter in lung parenchyma than the slow progressors, particularly for the CD20+ cells per square millimeter which were twice as numerous in the rapid progressors (**Table 4**). All inflammatory cells per square millimeter correlated negatively with the FVC % predicted at diagnosis in the end-stage slow progressors (CD45+: *r* = -0.37, *p* = 0.05; CD8+: *r* = -0.51, *p* = 0.006; CD4+: *r* = -0.42, *p* = 0.028; CD20+: *r* = -0.39, *p* = 0.04) but not in the rapid progressors.

**Table 4:** Pathological characteristics of end-stage IPF categorized as slow and rapid progressor.

	Slow progressor (n=27)	Rapid progressor (n=12)	P Values
**TLS, n/cm^2^**	6.58 (0–19.7)	6.3 (1.7–14.1)	n.s.
**TLS mean area, mm^2^**	0.033 (0.0029–0.058)	0.027 (0.014–0.063)	n.s.
**TLS activation score, %**	73 (7.1–94)	73.8 (43.75–87.5)	n.s.
**CD45/mm^2^**	370 (149–766)	427 (333–789)	*P* = 0.05
**CD8/mm^2^**	38 (7.6–196)	67 (26–174.7)	*P* < 0.01
**CD4/mm^2^**	114 (20–563)	190.5 (112–284)	*P* < 0.03
**CD20/mm^2^**	42 (3.4–389)	90.45 (51–107.7)	*P* = 0.007

Values are expressed as median and (range) or numbers and (%), as appropriate.

n.s.: non-significant.

TLS number, size, and activation score (**Table 4**) were similar in the slow and rapid progressors, but the TLS activation score correlated with the FVC decline over the time of follow-up in the rapid progressors (*r* = 0.73, *p* = 0.007; **Figure 4**) but not in the slow progressors (*r* = 0.1, *p* = 0.6), a result which might indicate a different and additional role of the TLS activity in the rapid progressors compatible with an autoimmune reaction (12).

## Discussion

A role for immune inflammation and autoimmunity, with a B-cell protagonist, has been proposed as an important factor in the pathogenesis of IPF (12, 25). This possibility conflicts with the prevailing concept that a diffuse inflammation and the presence of TLS ought to suggest an idiopatic interstitial pneumonias other than IPF (1, 20, 21).

In an attempt to clarify this point, we morphometrically quantified the number of TLS and inflammatory cells in the biopsied lungs of early IPF and in the explanted lungs of advanced IPF never treated with antifibrotic agents. We showed that TLS were present in all cases of early and end-stage IPF and that the total TLS area and degree of activation, assessed by the presence of CD40, were higher in late than in early disease. Furthermore, a prominent immune inflammatory infiltrate in the lung was already present in early IPF and persisted at end-stage, findings that are in line with the possible role of autoimmunity in the development of IPF (12).

Organized TLS, usually found in autoimmune diseases, mostly develop in the context of a chronic inflammation and in response to recognition of self-antigens, contributing to maintain the disease process and being associated with disease severity (14, 15, 26, 27). The frequency of TLS in autoimmune diseases varies from a minority of patients with systemic lupus erythematosus to 100% of those with thyroiditis (14). It is interesting to note that, in our population with well-established IPF, 100% of early and end-stage cases had well-formed TLS which increased in area and degree of activation from the early to the end stage of the disease. TLS in IPF, functioning as germinal centers, are potentially capable of sustaining B-cell differentiation and clonal selection toward autoantigens, which are frequently the target of autoantibodies detectable in patients with IPF (12).

The presence of adaptive inflammation and TLS in the early stages of IPF suggests that the immune inflammatory process is an important mechanism from the beginning of the disease, while the increased TLS area and degree of activation in late stage suggest that disease progression is correlated to the increase in TLS activation.

An inflammatory reaction precedes and leads to fibrosis in the bleomycin mouse model (28), whereas in human IPF the presence of an early inflammatory phase is not clearly established. Our results demonstrating an important lung inflammation and the presence of TLS in biopsies of early IPF indicate that inflammation might be playing an important role from the beginning of the disease, a finding that recalls the recent description of T- and B-cell infiltration and TLS in minimal fibrotic regions of the lung of IPF (11). Importantly, in our whole population, the number of inflammatory cells in the lung correlated positively with the number of TLS and negatively with the FVC at diagnosis.

The parenchymal inflammation in the early phases of IPF is characterized by an adaptive immune infiltrate with CD4+T cells, CD8+T cells, and B cells similar to that present in end-stage IPF, except for the CD8+T cells that were higher in the early stages. In our experience, CD4+T cells tend to aggregate not only near lymphoid follicles, as it has been reported (29), but also diffusely throughout the lung parenchyma. It is interesting to note that the high concentrations of IL-4 and IL-13 observed in BAL of IPF patients and also in mouse models point toward a CD4+T cell with a Th2 predominance, which would favor a profibrotic reaction (29). Similarly, CD8+T cells found diffusely throughout the parenchyma (19, 29) have been described as having a profibrotic/proinflammatory profile (29). The negative correlations between lung CD8+T cells and FVC at presentation in our study and also in Daniil et al. (30) point to their role in the development and progression of IPF. CD20+B cells were present not only in cohesive focal clusters forming TLS but also diffusely throughout the lung parenchyma both in early and late disease. Along with our findings is the recent morphometrical analysis of IPF lungs showing an important correlation of the degree of inflammation of T and B cells and TLS with the degree of fibrosis density (10).

IPF is defined as a progressive disease, with the majority of subjects experiencing a slow but steady worsening (slow progressors), while others have an accelerated decline (rapid progressors) (1). The findings in our study could shed some light into this divergent behavior. The inflammatory infiltrate in the lung parenchyma is probably important in the outcome of the slow progressors, as shown by the negative relation with the FVC at diagnosis. By contrast, in the rapid progressors, TLS activity score correlated with FVC decline during the time of follow-up, a finding not seen in the slow progressors. Furthermore, the number of B cells in the lung parenchyma was twice as high in the rapid than in the slow progressors, findings suggesting a different immunological response in the two groups of patients. The observed association of the rapid progression with TLS activation and B cell infiltration could be the immunopathological bases for the newly described generation of autoantibodies to periplakin (PPL), which can exacerbate a profibrotic/inflammatory response associated with a worse outcome (12).

The mechanism responsible for the preferential accumulation of autoreactive B cells in TLS in immune-derived disease is not fully understood, but a direct role has been proposed for Epstein–Barr virus (EBV) (31) in association with the presence of the HLA-DRB1-15:01 class II allele (31–34). Both EBV and HLA-DRB1-15:01 class II allele have been shown to be present in cases with IIP and IPF (26, 35–37). This allele acts as a coreceptor for EBV entry into B cells, favoring their transit to TLS where they differentiate to high-affinity autoreactive plasma cells (31). It is interesting to note that, in a disease with immunological similarities with IPF like multiple sclerosis, the association of persistent EBV in B cells with the presence of the HLA-DRB1-15:01 class II allele (31, 34) is an important risk factor for the development of the disease. These findings invite the speculation that the pairing of EBV with HLA-DRB1-15:01 might have some role in the mechanism of IPF rapid progressor phenotype.

Recently, data from INPULSIS and INBUILD (2, 3) showed that progressive ILDs have clinical courses and treatment responses similar to IPF, irrespective of the underlying ILD diagnosis and pattern on HRCT. Accordingly, the INBUILD authors stated: “these data support the hypothesis that progressive fibrosing interstitial lung diseases, regardless of clinical diagnosis, have a similar pathobiological mechanism” (3). The results of our and other studies (10, 11), showing a prominent B cell inflammation with activated TLS as features of IPF, similarly to other ILDs, might represent the common bases for the lung response to a variety of profibrotic insults and support the notion that a persistent adaptive immune response contributes to the fibrotic remodeling process in IPF. The presence of lung B-cell infiltration, activated TLS, and autoantibodies to PPL and other autoantigens (12) suggests that the use of anti-CD20 monoclonal antibodies like rituximab or ocrelizumab (38, 39), now being used in non-IPF ILDs, could be effective in the treatment of IPF.

Our study has several limitations. Firstly, participant recruitment occurred prior to the latest guidelines, potentially leading to variations in case classification according to updated recommendations. Consequently, we cannot rule out that certain cases may be classified differently at present. To enhance the clarity and scientific robustness of our findings, future research could incorporate the utilization of more recent diagnostic tools or entail longitudinal studies encompassing a broader and more diverse participant cohort. We also acknowledge that studying different subjects to compare early with end-stage IPF could be a limitation to the study. However, our results are similar to those reported by Todd (17) who examined the same subjects at the beginning and at the end stage of the disease. Our data are necessarily mainly descriptive but, having the unique opportunity to study lungs never treated with antifibrotics, have provided an accurate account of the inflammation, TLS presence and activity, and their likely role in IPF physiopathology.

In conclusion, both TLS and an adaptive immune inflammation with prominent B-cell participation are important components of IPF pathology from the beginning of the disease, likely playing an important role on its mechanism and progression. These results suggest that therapies guided toward the control of the B-cell function might prove to be beneficial.

## Data Availability

The original contributions presented in the study are included in the article/supplementary material. Further inquiries can be directed to the corresponding author.
