# Supplementary material for: Tertiary lymphoid structures and B-cell infiltration are IPF features with functional consequences
Source: Front Immunol. 2024 Oct 11;15:1437767. doi: 10.3389/fimmu.2024.1437767 (PMC11502372; doi:10.3389/fimmu.2024.1437767)
Supplement: Supplementary file 1 [file DataSheet1.docx]

**Supplementary material**

**Tertiary Lymphoid Structures and B-cell infiltration are IPF features with functional consequences**

Elisabetta Cocconcelli*^1^, Elisabetta Balestro*^1^, Graziella Turato^1^, Giordano Fiorentù^1^, Erica Bazzan^1^, Davide Biondini^1,2^, Mariaenrica Tinè^1^, Nicol Bernardinello^1^, Federica Pezzuto^1^, Simonetta Baraldo^1^, Fiorella Calabrese^1^, Federico Rea^1^, Alessandro Sanduzzi Zamparelli^3^, Paolo Spagnolo^1^, Manuel G Cosio†^1,4^, Marina Saetta†^1^

1. Department of Cardiac, Thoracic, Vascular Sciences and Public Health, University of Padova, Padova, Italy

2. Department of Medicine, University of Padova, Padova, Italy

3. Respiratory Medicine Unit at the Monaldi Hospital, AO dei Colli, Department of Clinical Medicine and Surgery, Federico II University, Naples, Italy

4. Meakins-Christie Laboratories, Respiratory Division, McGill University, Montreal, Quebec, Canada

* These authors contributed equally to this work.

† These authors contributed equally to this work.

**Pathological analysis**

Pathological analysis was performed by immunohistochemistry and light microscopy using a light microscope (Leica DMLB; Leica, Cambridge, UK) connected to a video recorder linked to a computerized image system (Software: Leica Application Suite). The cases were coded, and the measurements made without knowledge of clinical data.

Interstitial inflammation in the 3 groups of subjects (early IPF, end stage IPF and controls) was characterized quantifying B lymphocyte (CD20+), T lymphocytes (CD4+ and CD8+) and total leukocytes (CD45+) as previously reported (reference S1).

Briefly, each inflammatory cell type was quantified in 20 non consecutive high power fields per slide, counting inflammatory cells in one field, overlooking the three consecutive fields and counting the fourth one. Results were expressed as number of positive cells per mm^2^ of lung parenchyma examined.

To investigate TLS activation, the TLS expression of the co-stimulatory and activation marker CD40 [reference S2] was examined using a semiquantitative score.

To better characterize TLFs, CD4+ and CD8+ T Lymphocytes were quantified also inside the TLFs, in the area around the B cell germinal center (CD20+) and expressed as CD4/CD8 ratio (Figure S1).

All inflammatory cells were diffusely present throughout the lung parenchyma (Fig.S2).

**Figure S1.** CD4+/CD8+ T lymphocytes ratio in lymphoid follicles and in lung parenchyma in *early* IPF, *end stage* IPF and smoking controls. Bars indicates the median value.

**Figure S2.** Inflammatory cells in lung tissue. Microphotographs showing CD4+ (A), CD8+ (B) T cells and CD20+ (B) B cells in lung tissue of an end-stage IPF. Scale bar: 50 um.

Details on antibodies and detection systems are reported in Table A.

Table A: Immunohistochemistry

| Antibody | Dilution | Antigen retrieval method | Antigen detection |
| --- | --- | --- | --- |
| Mouse monoclonal anti-CD45 (DAKO  M 0701) | 1:60 | Microwave heat in citrate buffer pH 6 | Anti-mouse immunoglobulins coniugated with alkaline phosphatase and liquid permanent red |
| Mouse monoclonal anti-CD4 (DAKO  M 7310) | 1:50 | Microwave heat in EDTA buffer pH 8 | Envision detection system, peroxidase and diaminobenzidine (DAB) |
| Mouse monoclonal anti-CD8 (DAKO  M 7103) | 1:50 | Microwave heat in citrate buffer pH 6 | Anti-mouse immunoglobulins coniugated with alkaline phosphatase and liquid permanent red |
| Mouse monoclonal anti-CD20 (DAKO  M 0755) | 1:100 | Microwave heat in citrate buffer pH 6 | Envision detection system, peroxidase and diaminobenzidine (DAB) |
| Rabbit policlonal anti-CD40 (INVITROGEN) | 1:100 | Microwave heat in | Envision detection system, peroxidase and diaminobenzidine (DAB) |

**References**

S1. Balestro E, Calabrese F, Turato G, et al. Immune Inflammation and Disease Progression in Idiopathic Pulmonary Fibrosis. PLoS One. 2016;11:e0154516.

S2. Elgueta R, Benson MJ, de Vries VC, Wasiuk A, Guo Y, Noelle RJ. Molecular mechanism and function of CD40/CD40L engagement in the immune system. Immunol Rev. 2009;229:152-72
